# *Dictyosphaerium*‐like morphotype in terrestrial algae: what is *Xerochlorella* (Trebouxiophyceae, Chlorophyta)?^1^

**DOI:** 10.1111/jpy.12974

**Published:** 2020-03-16

**Authors:** Tatiana Mikhailyuk, Andreas Holzinger, Petro Tsarenko, Karin Glaser, Eduard Demchenko, Ulf Karsten

**Affiliations:** ^1^ M.G. Kholodny Institute of Botany National Academy of Sciences of Ukraine Tereschenkivska Str. 2 Kyiv 01004 Ukraine; ^2^ Functional Plant Biology Department of Botany University of Innsbruck Sternwartestrasse 15 A‐6020 Innsbruck Austria; ^3^ Applied Ecology and Phycology Institute of Biological Sciences University of Rostock Albert‐Einstein‐Strasse 3 D‐18059 Rostock Germany

**Keywords:** Chlorophyta, *Dictyosphaerium*, epitypification, integrative approach, phylogeny, taxonomy, ultrastructure, *Xerochlorella*

## Abstract

Several strains of terrestrial algae isolated from biological soil crusts in Germany and Ukraine were identified by morphological methods as the widely distributed species *Dictyosphaerium minutum* (=*Dictyosphaerium chlorelloides*). Investigation of the phylogeny showed their position unexpectedly outside of Chlorellaceae (Trebouxiophyceae) and distantly from *Chlorella chlorelloides,* to which this taxon was attributed after revision of the genus *Chlorella* based on an integrative approach. SSU rRNA phylogeny determined the position of our strains inside a clade recently described as a new genus of the cryptic alga *Xerochlorella olmiae* isolated from desert biological soil crusts in the United States. Investigation of the morphology of the authentic strain of *X. olmiae* showed *Dictyosphaerium*‐like morphology, as well as some other characters, common for our strains and morphospecies *D. minutum*. The latter alga was described as terrestrial and subsequently united with the earlier described aquatic representative *D. chlorelloides* because of their similar morphology. The revision of *Chlorella* mentioned above provided only one aquatic strain (*D. chlorelloides*), which determined its position in the genus. But terrestrial strains of the morphospecies were not investigated phylogenetically. Our study showed that the terrestrial *D. minutum* is not related to the morphologically similar *D. chlorelloides* (=*Chlorella chlorelloides*, Chlorellaceae), and instead represented a separate lineage in the Trebouxiophyceae, recently described as genus *Xerochlorella*. Therefore, revision of *Xerochlorella* is proposed, including nomenclatural combinations, epitypifications, and emendations of two species: *X. minuta* and *X. dichotoma*. New characters of the genus based on investigation of morphology and ultrastructure were determined.

**Abbreviations**
AIC: Akaike information criterion
KW: Herbarium of the M.G. Kholodny Institute of Botany of the National Academy of Sciences of Ukraine
ML: maximum likelihood
SAG: Culture Collection of Algae at Göttingen University, Germany
UTEX: Culture Collection of Algae at the University of Texas at Austin

Development of modern taxonomy of microalgae proceeds on the basis of wide usage of molecular‐phylogenetic methods. These methods are powerful tools to determine with high accuracy relationships between organisms, their phylogenetic position among the numerous groups of algae, and to identify morphologically cryptic taxa (Leliaert et al. 2014). Since many microalgae are organisms with a very limited number of morphological characters for taxonomic purposes (because of simple morphology and life cycle, high adaptive and morphological plasticity, etc.) molecular‐phylogenetic methods often are the main or even only tool to delimit taxa. Therefore, algal identification based on molecular markers (including culture independent methods, environmental, and next generation sequencing) represents the current state of the art approach (De Clerck et al. 2013, Leliaert et al. 2014, Zimmermann et al. 2015, Büdel et al. 2016).

Despite fast development of new methods and their wide practical application, the main part of accumulated knowledge of algae of different taxonomic groups, such as their diversity, distribution, ecology is still based on the classical morphological approach developed during the middle of 19th century (Friedl and Rybalka 2012, De Clerck et al. 2013, Ettl and Gärtner 2014, Büdel et al. 2016 etc.). Many algal species were originally described based on field material or algal cultures, both of which were later lost in many cases. The type information of these species was usually preserved as line drawings. Further investigation of lost type material by genetic methods is possible only by designating a reference strain as an epitype, with morphological and ecological characters as well as geographical distribution corresponding as close as possible to the original description of the respective taxon.

The so‐called integrative approach (combination of classical algal cultivation and microscopy with new molecular‐phylogenetic methods) is a feasible instrument to combine morphological and genetic information, particularly for biodiversity studies (Darienko et al. 2015). There exists now many papers devoted to taxonomic revisions of green, streptophycean, xanthophycean, eustigmatophycean algae, cyanobacteria, etc., using such an integrative approach (Neustupa et al. 2011, Rybalka et al. 2013, Bohunická et al. 2015, Škaloud et al. 2016, Darienko et al. 2017, Kryvenda et al. 2018, Mikhailyuk et al. 2018, Darienko and Pröschold 2019 etc.).

Incompleteness of the algal genetic database sometimes leads to the fact that some newly discovered lineages may be described as new taxa. But in fact these algae were found and described by classical methods many years ago. The reasons for this problem are also the simplicity and plasticity of microalgal morphology. Using an integrative approach including molecular‐phylogenetic and follow‐up detailed morphological investigations of these taxa may show such disagreements.

An example of such taxonomical disagreement is the recently described desert green algal genus *Xerochlorella* (Fučíková et al. 2014). This is an authospore‐forming alga with small spherical cells (max 10 µm in diameter) and inconspicuous *Chlorella*‐like morphology. More detailed investigation of an authentic strain of this monotypic genus as well as several newly isolated strains led to the conclusion that *Xerochlorella* is identical to the widely distributed terrestrial species *Dictyosphaerium minutum*, described more than 80 years ago by classical methods (Petersen 1932, Ettl and Gärtner 2014). Therefore, this paper is devoted to the respective taxonomic revision of the genus *Xerochlorella* based on an integrative approach.

## Methods

### Strains, culture conditions, light microscopy, ultrastructure

As material for the present study, four unialgal original strains were used. These strains were isolated from terrestrial habitats (biological soil crusts from maritime sand dunes or forest soil) in Europe (Germany and Ukraine). The isolation procedure and culture conditions were described in a previous paper (Schulz et al. 2016). For comparison, an authentic strain of the recently described *Xerochlorella olmiae*, UTEX B 2993 (isolated from desert soil crust [USA]) was used. DNA sequences were compiled from GenBank of other *Dictyosphaerium*‐like strains phylogenetically related to *Xerochlorella*. Short information about all strains and sampling sites are provided in Table 1.

**Table 1:** Summarized information about strains and sequences of *Xerochlorella* used in the present study (newly obtained sequences are marked with Bold)

Original species name	Strain label/culture number	Collection information	Species designation	SSU rRNA	ITS‐1–5.8S rRNA–ITS‐2
*Xerochlorella olmiae*	UTEX B 2993	Mojave National Preserve, San Bernardino Co., California, USA, Desert soil crust 35°27.113′ N, 115°40.550′ W, Louise A. Lewis (2003)	*Xerochlorella minuta*	**MN267184**	
*Xerochlorella olmiae*	BCP‐EM3VF21	Mojave National Preserve, San Bernardino Co., California, USA, Desert soil crust 35°27.113′ N, 115°40.550′ W, Louise A. Lewis (2003)	*Xerochlorella minuta*	KF693788	–
*Dictyosphaerium* sp.	UTEX SNO65	Antarctic (?)^a^	*Xerochlorella minuta*	GQ502290	
*Dictyosphaerium* sp.	CCAP 222/3	Moss epiphyte, Signy Island, South Orkney Islands, Antarctica, Broady (1975) as *Dictyosphaerium chlorelloides* (formerly listed as *Dictyosphaerium minutum*)	*Xerochlorella minuta*	GQ502289	
*Dictyosphaerium minutum*	CCAP 222/3	Moss epiphyte, Signy Island, South Orkney Islands, Antarctica, Broady (1975) as *Dictyosphaerium chlorelloides* (formerly listed as *Dictyosphaerium minutum*)	*Xerochlorella minuta*	FR865691	
*Dictyosphaerium* sp.	CCALA 333	Slovakia, Vysoke Tatry, peat bog, periphyton Ruzicka, 1962, listed as *Dictyosphaerium tetrachotomum* Printz	*Xerochlorella minuta*	GQ487247	–
*Dictyosphaerium chlorelloides*	Us‐7‐12	Baltic sea coast, sand dunes, soil crust, Zempin, Usedom, Mecklenburg‐Vorpommern, Germany, 54°04.172′N; 13°58.035′E, T. Mikhailyuk, 2013	*Xerochlorella minuta*	MH703761	
*Dictyosphaerium chlorelloides*	SEW‐9‐1	Soil crust, beech forest, Germany, 53°02.674′N; 13°48.617′E, K. Glaser and T. Mikhailyuk, 2014	*Xerochlorella minuta*	**MN267183**	
*Dictyosphaerium chlorelloides*	Prim‐17‐2	Black Sea coast, sand dunes, Danube Delta Biosphere Reserve, Zhebryianska bay, Kiliya District, Odessa Region, 45.486662736 N; 29.633074533 E, Demchenko and T. Mikhailyuk, 2013	*Xerochlorella minuta*	**MN267182**	
*Dictyosphaerium dichotomum*	Hg‐2‐3 SAG 2582	Baltic sea coast, sand dunes, soil crust, Heiligendam, Mecklenburg‐Vorpommern, Germany, 54.146102096 N, 11.86013389 E, T. Mikhailyuk, 2013	*Xerochlorella dichotoma*	**MN267185**	

a Information concerning this strain is from NCBI, but the information from UTEX catalogue is different: *Chloromomas rosae*, Litchfield Island, Antarctic, red ice*,* Collection: B. Bidigare (3/26/90), Isolation: R.W. Hoham.

Purified unialgal strains were maintained on solid medium (1.5% agar with 3N BBM and vitamins; Starr and Zeikus 1993) at 20°C with 25 μmol photons · m^−2^ · s^−1^ (Osram Lumilux Cool White lamps L36W/840) under a light/dark cycle of 12:12 h light:dark. Morphological examination of these unialgal cultures was performed using Olympus BX51 and Zeiss Axiovert 200 M light microscopes with Nomarski DIC optics. Photomicrographs were taken with digital cameras [Olympus UC30 (Tokyo, Japan) and Zeiss Axiovision (release 4.7, Oberkochen, Germany)] attached to the respective microscopes and processed by software cell Sens Entry and Zeiss Axiovision.

Samples were fixed for transmission electron microscopy (TEM) using a standard chemical fixation protocol (2.5% glutaraldehyde, 1% OsO_4_ in 10 mM caccodylate buffer, pH = 6.8) according to Holzinger et al. (2009). Samples were dehydrated in increasing ethanol concentrations, transferred to modified Spurr's resin and heat polymerized. For TEM, ultrathin sections were prepared, counterstained with uranyl acetate and Reynold's lead citrate, and investigated using a Zeiss LIBRA 120 transmission electron microscope at 80 kV. Images were captured with a TRS 2k SSCCD camera and further processed using Adobe Photoshop software (Adobe Systems Inc., San José, CA, USA).

### DNA isolation, PCR and sequencing

Genomic DNA of all investigated strains was extracted using the DNeasy Plant Mini Kit (Qiagen GmbH, Hilden, Germany) according to the manufacturer's instructions. Nucleotide sequences of the SSU rRNA gene together with ITS‐1‐5.8S‐ITS‐2 region were amplified using a set of Taq PCR Mastermix Kit (Qiagen GmbH) and a complex of EAF3 and ITS055R as well as algal‐specific primers G800R and G500F. PCR reactions were made in a thermocycler T gradient Thermoblock (Biometra, Analytik Jena, Germany) under conditions described in a previous paper (Mikhailyuk et al. 2018). PCR products were cleaned using a Qiagen PCR purification kit (Qiagen GmbH) according to the manufacturer's instructions. Cleaned PCR products were sequenced commercially by Qiagen Company using primers G800R, 536R, 920F, 1400R, 1400F, GF, GR, and ITS2F. Sequences of all primers used in the study with respective references are included in the Table 2. The resulting sequences were assembled and edited using Geneious software (version 8.1.8; Biomatters). They were deposited in GenBank under accession numbers MN267182 – MN267185.

**Table 2:** List of primers used in the study for amplification and sequencing

Primer name	Sequence [5′‐>3′]	Reference
EAF3	TCGACAATCTGGTTGATCCTGCCAG	Marin et al. (2003)
ITS055R	CTCCTTGGTCCGTGTTTCAAGACGGG	Marin et al. (1998)
G500F	GAATGAGTACAATCTAAACCCCTTAAC	Darienko et al. (2019)
G800R	CATTACTCCGGTCCTACAGACCAACAGG	
536R	GWATTACCGCGGCKGCTG	Lane (1991)
920F	GAAACTTAAAKGAATTG	Hoef‐Emden and Melkonian (2003)
1400F	CTGCCCTTTGTACACACCGCCCGTC	
1400R	GGTAGGAGCGACGGGCGGTGTGTAC	Marin et al. (2003)
GF	GGGATCCGTTCCCGTAGGTGAACCTGC	Goff and Moon (1993)
GR	GGGATCCATATGCTTAAGTTCAGCGGGT	
ITS2F	GCATCGATGAAGAACGCAGC	White et al. (1990)

### Phylogenetic analyses

DNA sequences of our isolates were compared to those from reference strains at NCBI using BLASTn queries (http://blast.ncbi.nlm.nih.gov). For comparison with original strains, we used nucleotide sequences available in GenBank (NCBI) of representatives of the Trebouxiophyceae, with selected Chlorophyceae as the outgroup. Multiple alignment of the nucleotide sequences of the SSU rRNA was made using MAFFT web server (version 7; Katoh and Standley 2013) followed by manually editing in the program BioEdit (version 7.2). Alignment for the phylogeny of the ITS‐2 region was performed manually in BioEdit, taking into account the secondary structure of the RNA (see below).

The evolutionary model that is best suited to the database was selected on the basis of the lowest AIC value (Akaike 1974) and calculated in MEGA (version 6; Tamura et al. 2013). Phylogenetic trees were constructed in the program MrBayes 3.2.2 (Ronquist and Huelsenbeck 2003), using an evolutionary model GTR + G + I, with 5,000,000 generations. Gaps were treated as missing character. Two of the four runs of Markov chain Monte Carlo were made simultaneously, with the trees, taken every 500 generations. Split frequencies between runs at the end of calculations were below 0.01. The trees selected before the likelihood rate reached saturation were subsequently rejected. The reliability of tree topology verified by the maximum likelihood analysis (ML, GTR + I + G) was made using the program GARLI 2.0, and bootstrap support was calculated with 1,000 replicates.

### Analysis of the ITS‐2 secondary structures, genetic similarity

Models of the secondary structure of ITS‐2 together with 5.8S‐LSU rRNA stem were predicted for all investigated strains of *Xerochlorella*. Helices were folded with the online software Mfold (Zuker 2003) and visualized in the online tool PseudoViewer (Byun and Han 2009). ITS‐2 secondary structure of CCAP 222/3 was predicted based on of two published sequences with different length (GQ502289 and FR865691). A part of 5.8S‐LSU stem and neighboring core of UTEX SNO65 are unknown (because of short sequence (GQ502290) and therefore could not be analyzed. Compensatory base changes (CBCs and hemi‐CBCs) as well as mismatches, deletions, single, or unpaired bases were estimated using recommendations published in Demchenko et al. (2012).

Genetic similarity of SSU and ITS rRNA between investigated strains was calculated in the program MEGA using p‐distance and uniform rates, and was expressed in percent.

## Results

### Molecular phylogeny based on SSU rRNA gene and ITS‐2

**Figure 1:**
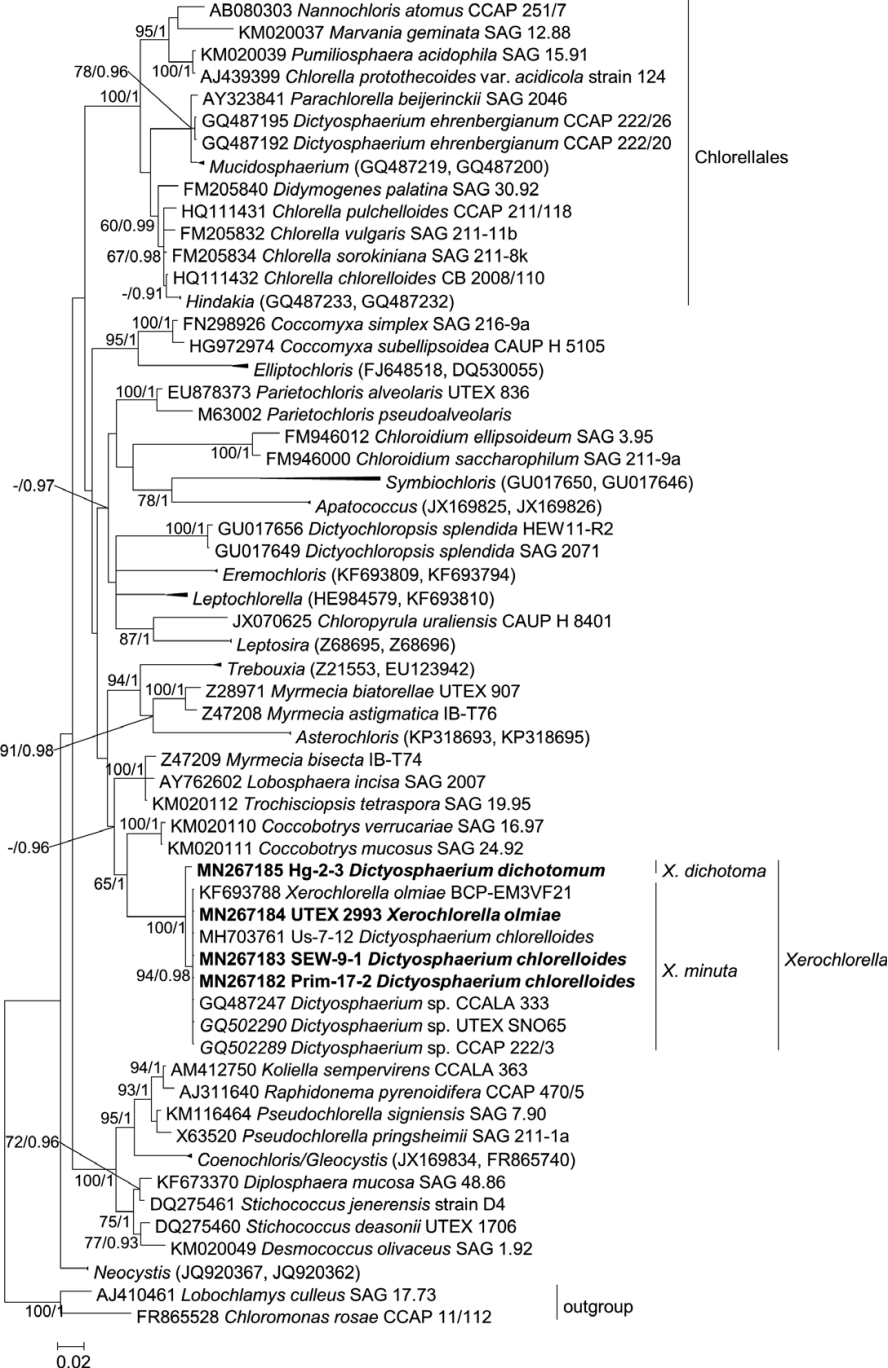
Molecular phylogeny of Trebouxiophyceae (Chlorophyta) based on the comparison of the nucleotide sequences of the SSU rRNA gene (1776 base pairs). A phylogenetic tree was inferred by the Bayesian method with Bayesian Posterior Probabilities (PP) and Maximum Likelihood bootstrap support (BP); PP values lower than 0.8 and BP lower than 50% not shown. Strains in bold represent newly sequenced algae. Clades were named according to Fučíková et al. (2014) and Bock et al. (2011b). Scale bar: 0.02 substitutions/site.

The phylogenetic analysis of SSU rRNA sequences revealed that the newly sequenced original isolates as well as original sequence of UTEX B 2993 clustered together with the authentic strain of *Xerochlorella olmiae* (BCP‐EM3VF21 = UTEX B 2993), as well as with several strains from CCAP, UTEX, and CCALA initially identified as *Dictyosphaerium* species (Fig. 1). All of these strains formed a highly supported *Xerochlorella* clade sister to the clades *Lobosphaera* and *Coccobotrys*, based on similar, almost identical SSU sequences, with exception of Hg‐2‐3 which formed a separate, highly supported lineage inside the *Xerochlorella* clade. This strain differed also morphologically (see below).

**Figure 2:**
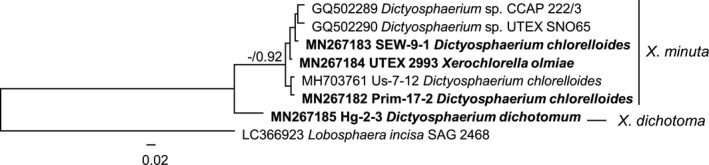
Molecular phylogeny of *Xerochlorella* (Trebouxiophyceae, Chlorophyta) based on the comparison of the nucleotide sequences of the ITS‐2 region (687 base pairs). A phylogenetic tree was inferred by the Bayesian method with Bayesian Posterior Probabilities (PP) and Maximum Likelihood bootstrap support (BP); PP values lower than 0.8 and BP lower than 50% not shown. Strains in bold represent newly sequenced algae. Scale bar: 0.02 substitutions/site.

To obtain better resolution within the *Xerochlorella* clade, a phylogenetic analysis of the ITS‐2 region of all investigated strains with *Lobosphaera* as outgroup was performed (Fig. 2). The tree confirmed also two main lineages inside the *Xerochlorella* clade: strains designated as *X. minuta* comb. nova (see below) and the morphologically different strain Hg‐2‐3 identified as *X. dichotoma* comb. nova.

### Comparison of ITS‐2 secondary structures, p‐distance

**Figure 3:**
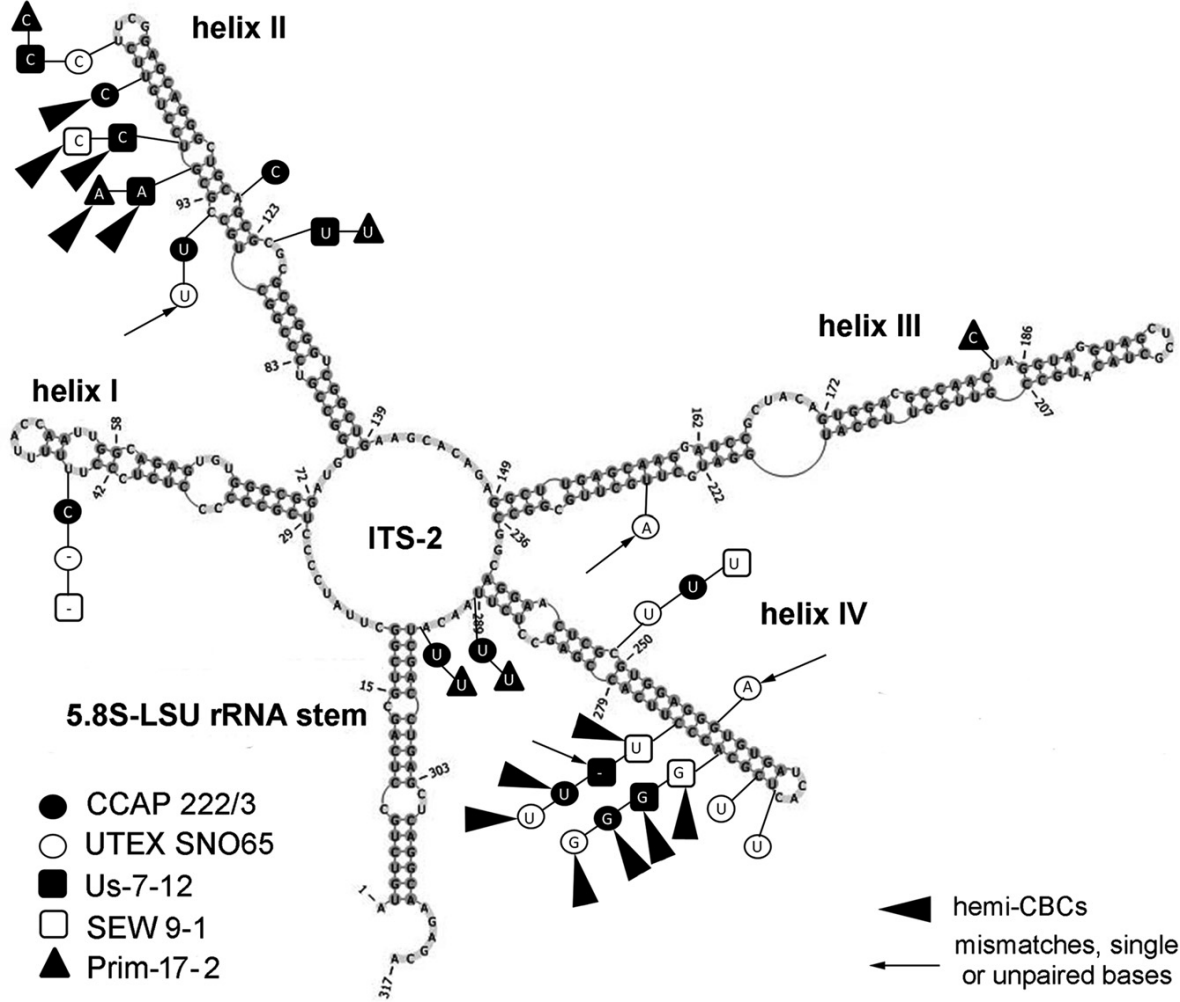
Comparison of ITS‐2 secondary structure of *Xerochlorella minuta* strains. The structure of the reference strain (UTEX B 2993) is presented with the marked differences to other strains of the species. Variable bases or basepairs are shown with circles, boxes, and triangles.

**Figure 4:**
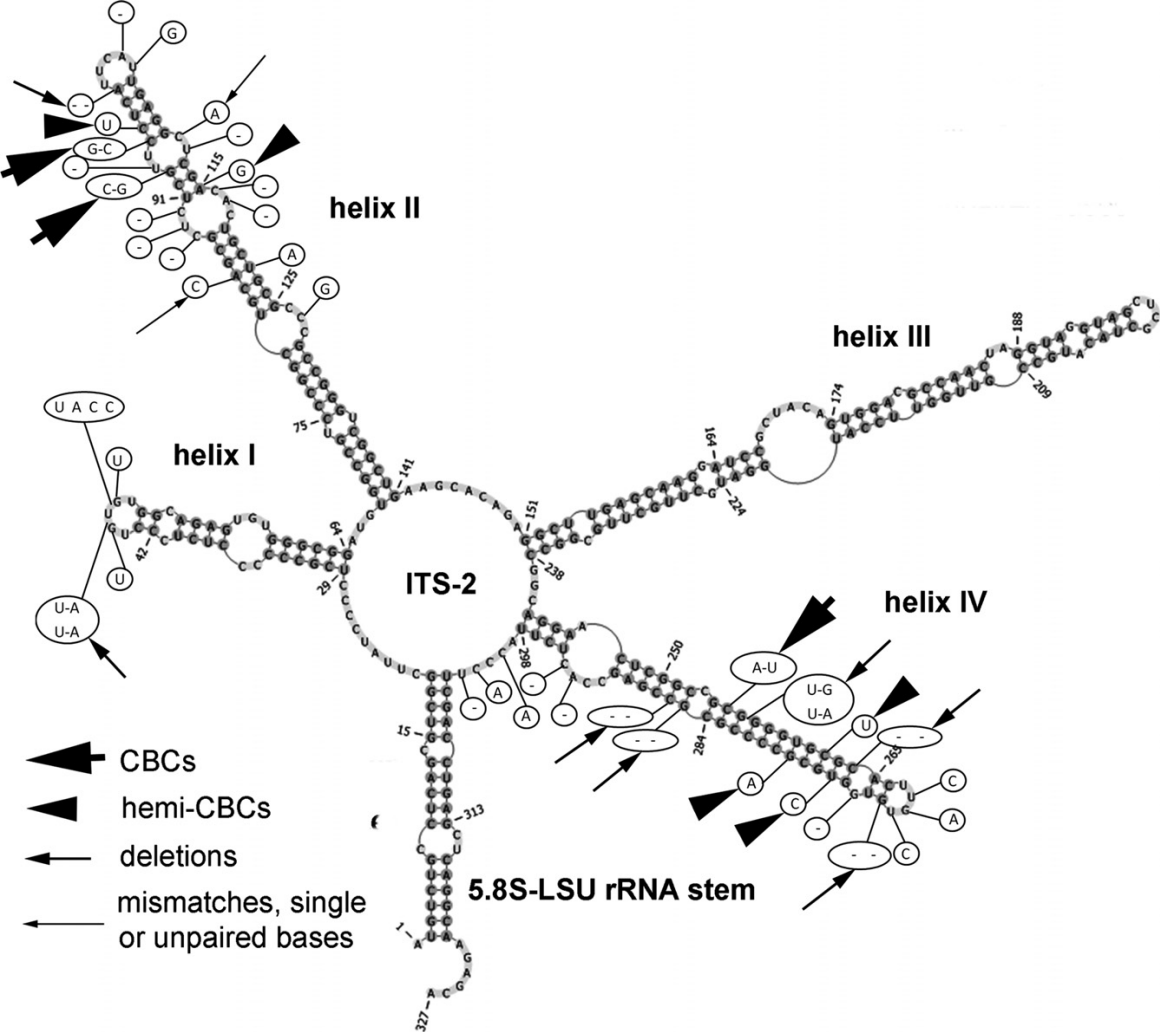
Comparison of ITS‐2 secondary structure of *Xerochlorella* species. The structure of *X. dichotoma* (reference strain Hg‐2‐3) is presented with the differences to *X. minuta* (reference strain UTEX B 2993). Variable bases or basepairs are shown with circles.

To evaluate borders between different species inside the *Xerochlorella* clade the secondary structure of ITS‐2 of all investigated strains was evaluated. Comparison of ITS‐2 secondary structure of strains referred to *X. minuta* (see below) showed generally high similarity (Fig. 3). The most striking differences were localized in helices II and IV, while the 5.8S‐LSU rRNA stem was identical in all strains. But many more differences in ITS‐2 secondary structure were found between these strains and the strain Hg‐2‐3 (Fig. 4). All differences were localized in helices I, II, and IV. Results of analysis of secondary structures of all strains are summarized in Table 3. No compensatory base changes (CBCs) between strains referred to *X. minuta* were found, while differences between these strains varied from 1 to 3 hemi‐CBCs, 1–4 mismatches and 2–8 nucleotide differences, all of which were localized in loops or ITS‐2 core (Fig. 3, Table 3). Differences between secondary structures of ITS‐2 of all *X. minuta* strains and Hg‐2‐3 were more prominent: 3–4 CBCs, 4–6 hemi‐CBCs, 9 deletions, 2–3 mismatches, and 25–28 nucleotide differences in loops or ITS‐2 core (Fig. 4, Table 3).

**Table 3:** Comparison of different strains of Xerochlorella by CBC approach and genetic similarity of SSU and ITS rRNA

	UTEX B 2993	CCAP 222/3	UTEX SNO65	Us‐7‐12	SEW‐9‐1	Prim‐17‐2	Hg‐2‐3
UTEX B 2993		‐/3/‐/‐/6	‐/3/‐/3/4	‐/3/‐/1/2	‐/3/‐/‐/2	‐/1/‐/‐/5	3/5/9/2/25
CCAP 222/3	99.8		‐/2/‐/3/3	‐/3/‐/1/8	‐/2/‐/‐/5	‐/4/‐/‐/7	3/4/9/2/26
UTEX SNO65	99.5	99.6		‐/3/‐/4/4	‐/2/‐/3/2	‐/4/‐/3/5	4/4/9/3/28
Us‐7‐12	99.8	99.8	99.6		‐/1/‐/1/4	‐/2/‐/1/3	4/4/9/3/27
SEW‐9‐1	99.8	99.8	99.5	99.9		‐/4/‐/‐/7	4/4/9/2/26
Prim‐17‐2	99.9	99.8	99.5	99.8	99.8		3/6/9/2/28
Hg‐2‐3	97.7	97.6	97.4	97.7	97.8	97.5	

1: CBCs/hCBCs/deletions/mismatches/differences in loops or ITS‐2 core; 2: genetic similarity of SSU and ITS rRNA (%)

Pairwise comparison of SSU and ITS rRNA sequences of all strains investigated within the *Xerochlorella* clade showed a close similarity among all isolates referred to *X. minuta* (see below; Table 3). The identity of nucleotides of SSU and ITS rRNA of these strains varied from 99.5% to 99.9%. But strain Hg‐2‐3 considerably differed from those mentioned strains: the identity of nucleotides of the respective region varied from 97.5% to 97.8%.

### Morphology and reproduction

**Figure 5:**
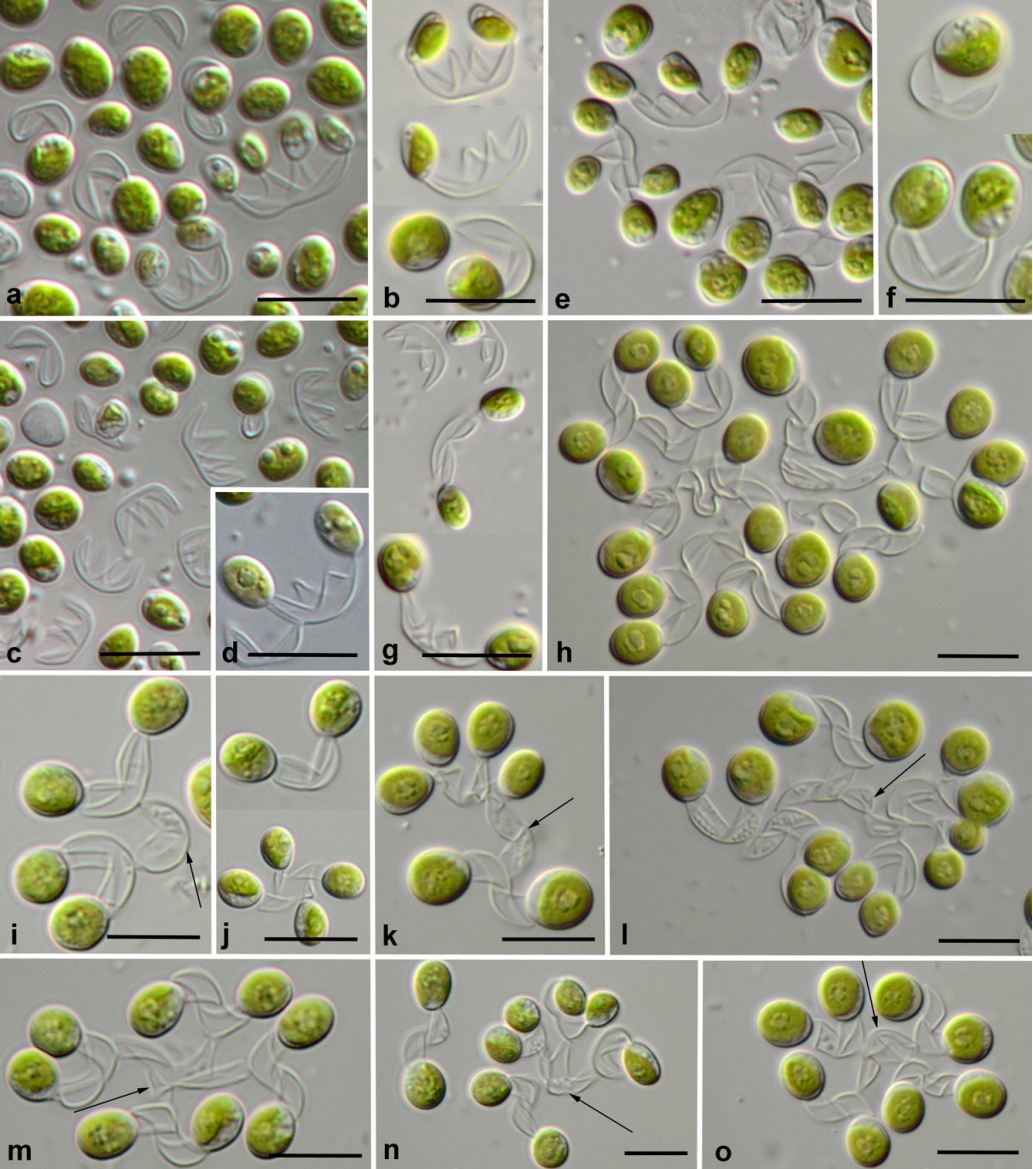
Morphology of *Xerochlorella* species: (a–g) *X. minuta*: (a–d) UTEX B 2993, (e, f) Us‐7‐12, (g) Prim‐17‐2. (h–o) *X. dichotoma*, Hg‐2‐3. (a, c, e) General view on culture material with small 2‐4‐celled colonies, fragments of sporangial walls and unicells. (b, f, g, j) Small 2‐4‐celled colonies. (h, i, k–o) Large colonies originated due to successive cell division from small two‐celled (i, k, l) and four‐celled colonies (m–o); initial fragments of sporangial walls showed with arrows. Scale bars: 10 μm.

The investigated strains represented unicellular green algae. Cells were small, ovoid to wide ellipsoid and almost spherical, (4.3)5.0–6.7(7.5) × (2.5)4.3–6.0(7.0) μm, uninucleate (Fig. 5). Cells of the strain Hg‐2‐3 were slightly larger, (4.5)6.1–8.1(9.6) × (3.9)5.7–7.8(8.6) μm. Chloroplasts were single, parietal, cup‐shaped, occupied half or 2/3 of the cells inner surface, with smooth or slightly waved margins. Pyrenoids were single, spherical to widely ellipsoid, in the middle of the chloroplast, surrounded by several starch grains. Nuclei were situated opposite the pyrenoid.

Cultures included single cells as well as cells united in colonies (Fig. 5, a and e). Colonies formed due to remnants of mother cell walls, which slightly gelatinized, but preserved in culture (Fig. 5c). Fragments of mother (sporangial) cell wall originated from sporangia with two autospores had semilunar shape (Fig. 5f). Sporangia with four autospores formed cruciform remnants (Fig. 5, b–d). Cells attached to the edges of remnants by their narrow ends. Therefore, semilunar (two‐celled) or cruciform (four‐celled) *Dictyosphaerium*‐like colonies were formed in the cultures (Fig. 5, a–g). A lot of remnants were distributed in culture without any cells, because autospores and young cells usually easily detached from remnants (Fig. 5c). These types of colonies and unicells were observed in the strains Us‐7‐12, SEW‐9‐1, Prim‐17‐2, and the authentic strain of *Xerochlorella olmiae* UTEX B 2993.

Strain Hg‐2‐3 formed similar colonies (Fig. 5j), but also much bigger cell aggregations especially prominent in older cultures. Large *Dictyosphaerium*‐like colonies formed due to tighter attaching cells to the fragments of sporangial walls than in other strains. Cells attached to the remnants formed sporangia and later their daughter cells released, but remained attached to remnants of sporangial walls. Therefore, subsequent division led to large colonies with cells attached to pseudodichotomous branching remnants of mother walls (Fig. 5, h, i, k–o).

**Figure 6:**
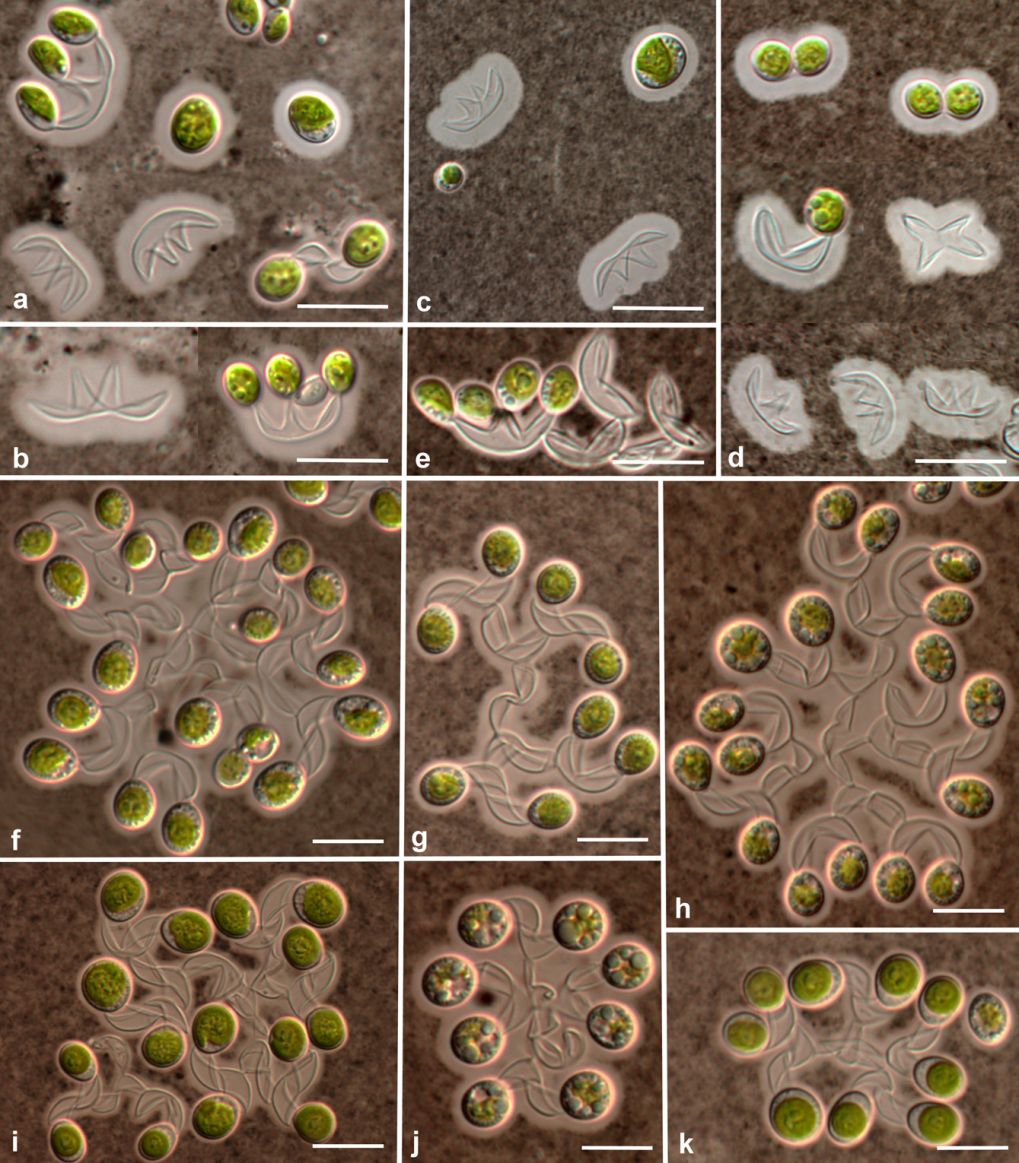
Negative staining of *Xerochlorella* species: (a–e) *X. minuta*, mucilage envelopes of different thickness around cells, fragments of sporangial walls and small 2‐4‐celled colonies: (a, b) UTEX B 2993, (c, d) Us‐7‐12, (e) Prim‐17‐2. (f–k) *X. dichotoma*, Hg‐2‐3, mucilage envelopes around colonies. Scale bars: 10 μm.

Small and large colonies of all strains were usually surrounded by a delicate layer of mucilage. Mucilage was visible after negative staining with Indian ink. The thickness of mucilage varied from 2.1 to 5.0 μm in the different strains (Fig. 6). Individual mucilages envelopes surrounding cells were also observed (Fig. 6, a–d).

Reproduction by autospores was observed with two or four autospores formed in sporangia. Sporangial walls ruptured, but remained in culture forming semilunar or cruciform remnants. Young cells usually attached to the remnants by their narrow ends, but also often detached and laid separately. The culture usually represented a mixture of unicells, remnants of mother cell walls and *Dictyosphaerium*‐like semilunar, cruciform or pseudodichotomous branching colonies depending on the strain (Fig. 5).

### Ultrastructure

**Figure 7:**
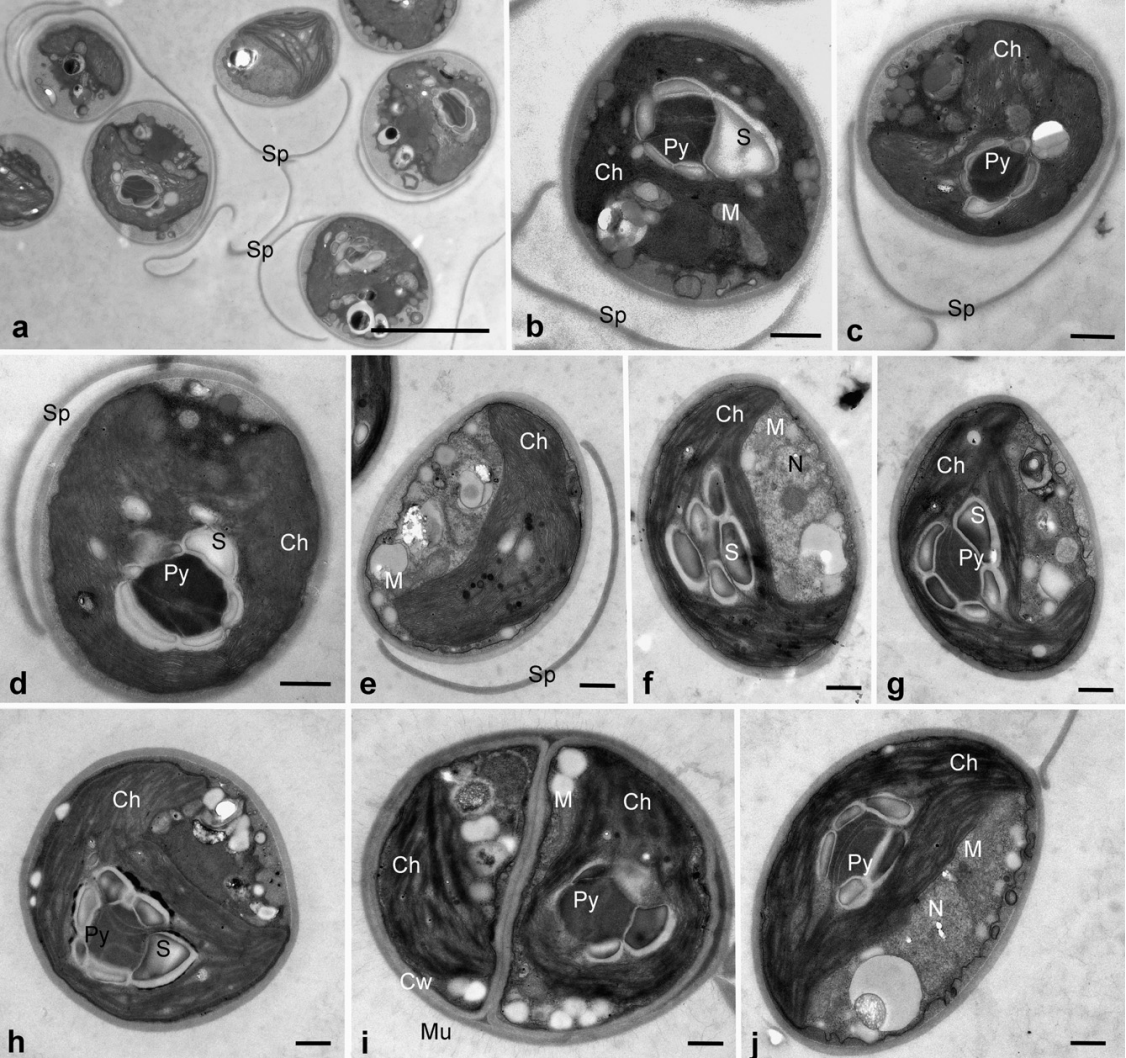
Transmission electron micrographs of vegetative cells of *Xerochlorella* species: (a–d) *X. minuta*, Us‐7‐12, (e–j) *X. dichotoma*, Hg‐2‐3. General view on culture with cells connected by fragments of sporangial walls (a), single cells (b–h, j) and sporangium with delicate mucilage envelope (i). Sp, fragments of sporangial walls, Ch, chloroplast; Py, pyrenoid; S, starch grains; N, nucleus; Cw, cell wall; Mu, mucilage envelope; M, mitochondrium. Scale bars: 5 μm (a) or 1 μm (b–j).

The ultrastructure was investigated in strains Us‐7‐12 and Hg‐2‐3. Generally it was similar (Fig. 7). The chloroplast was always located at one side of the cell covering half or 2/3 of cells’ inner surface and had a smooth or waved edges. The pyrenoid was surrounded by several (5–6) starch grains. The pyrenoid matrix was transversed by two to three thylakoid membranes. The nucleus was located opposite the pyrenoid (Fig. 7, f and j). Several mitochondrial profiles were observed in the cells, they were mostly located close to the chloroplast (Fig. 7, b, e, f, i and j). Cells were surrounded by a cell wall, and a delicate mucilage envelope was visible on some cells (Fig. 7i). A lot of remnants of cell walls were observed in the samples (Fig. 7, a–e).

## Discussion

### Phylogeny, SSU and ITS rRNA genetic similarity, ITS‐2 secondary structure and species delimitation within *Xerochlorella*

Phylogenetic analysis based on SSU rRNA sequences mostly corresponded with the phylogeny of the Trebouxiophyceae by Fučíková et al. (2014), Darienko et al. (2016), Hodač et al. (2016), Škaloud et al. (2016), and others. All investigated strains formed a highly supported clade assigned to the genus *Xerochlorella* with the closest genera *Coccobotrys* and *Lobosphaera* (Fučíková et al. 2014). Therefore, all investigated strains should be identified as *Xerochlorella* species. ITS‐2 phylogeny showed that *Xerochlorella* represents two main lineages: strains CCAP 222/3, UTEX SNO65, SEW‐9‐1, Us‐7‐12, Prim‐17‐2 together with the authentic strain UTEX B 2993 corresponded to the type species *X. olmiae* (*X. minuta* comb. nova, see below), while strain Hg‐2‐3 represented another species identified initially as *Dictyosphaerium dichotomum* (*Xerochlorella dichotoma* comb. nova, see below).

Comparison of ITS‐2 secondary structure and pairwise comparison of SSU and ITS rRNA sequences confirmed the presence of two lineages in *Xerochlorella*. All strains corresponding to *X. minuta* are characterized by minor differences (1–3 hemi‐CBCs and missing CBCs). Comparison of these strains with Hg‐2‐3 (*X. dichotoma*) showed conspicuous differences as reflected in 3–4 CBCs and 4–6 hemi‐CBCs depending on strain. Therefore, the presence of CBCs in ITS‐2, essential differences according SSU and ITS‐2 phylogeny as well as in morphology clearly indicate that this genus includes now two species: *X. minuta* and *X. dichotoma*.

### Definition of the genus *Xerochlorella*

*Xerochlorella* is a monotypic genus of green algae (Trebouxiophtyceae), which was recently described based on SSU rRNA and *rbc*L phylogenies and morphological investigation of two strains (Fučíková et al. 2014). The alga was found during investigation of desert biological soil crusts (Mojave National Preserve, San Bernardino Co., California, USA). It is characterized by simple *Chlorella*‐like morphology, small spherical or oviform cells and a chloroplast without pyrenoid. The chloroplasts of young cells have smooth margins, later dissects on two lobes and then on several separate chloroplasts. This representative formed a separate lineage in the phylogeny of Trebouxiophtyceae. The lineage also included the strain CCAP 222/3, isolated by P.A. Broady from Antarctic soil and deposited in a culture collection under the name *Dictyosphaerium minutum*. But this strain is non‐authentic, since the genus *Dictyosphaerium* forms another phylogenetic lineage within Trebouxiophyceae (Chlorellales) and *Dictyosphaerium*‐like morphology was not typical for strains isolated from desert soil. Therefore, the new genus *Xerochlorella* was proposed. Fučíková et al. (2014) mentioned in their paper that *X. olmiae* was possibly found earlier and perhaps described based on classical methods. But simple morphology does not allow to determine this taxon using light microscopy. Therefore, “…. knowing the limitations of the morphological approach, we prefer to establish a new taxon supported by an authentic strain, even though some of our newly proposed taxa could fit some of the uncertain historical morphogenera” (Fučíková et al. 2014).

The investigated strains (Us‐7‐12, SEW‐9‐1 and Prim‐17‐2) were characterized by *Dictyosphaerium*‐like morphology and were preliminary identified as *Dictyosphaerium chlorelloides* (=*Dictyosphaerium minutum*) based on the “Syllabus der Boden‐, Luft und Flechtenalgen” (Ettl and Gärtner 2014). This taxon was mentioned as *Chlorella chlorelloides* in some earlier papers (Schulz et al. 2016, Borchhardt et al. 2017a,b) taking into consideration the revision of Bock et al. (2011a), in which *D. chlorelloides* was assigned to the genus *Chlorella*. But molecular‐phylogenetic data obtained later indicated that our strains do not belong to *Chlorella* and Chlorellales (Mikhailyuk et al. 2019), instead assignment to *Xerochlorella* was proposed. Prominent *Dictyosphaerium*‐like morphology of the here investigated strains and the presence of several other strains preliminary identified as *Dictyosphaerium* in the *Xerochlorella* clade (see Fig. 1) indicated that the generic concept of *Xerochlorella* should be revised.

### *Dictyosphaerium* morphotype among green algae

The genus *Dictyosphaerium* (type species: *Dictyosphaerium ehrenbergianum*) unites mostly aquatic taxa characterized by a specific morphology: small *Chlorella*‐like cells are attached to variously branched mucilaginous stalks originating from remnants of mother (sporangial) cell walls (Komárek and Perman 1978). As a result colonies of different sizes surrounded by mucilage are formed. A molecular‐phylogenetic investigation of *Dictyosphaerium* showed that the *Dictyosphaerium*‐like morphotype is polyphyletic (Krienitz et al. 2010). Therefore, a position of *Dictyosphaerium* inside *Parachlorella* clade, Chlorellaceae (Bock et al. 2011b) was determined. Many morphospecies of *Dictyosphaerium* and *Pseudodictyosphaerium* were transferred to the genera *Chlorella* (Bock et al. 2011a) and *Mychonastes*, Chlorophyceae (Krienitz et al. 2011). Numerous new genera inside Chlorellaceae (*Mucidosphaerium,* Bock et al. 2011b; *Heynigia* and *Hindakia,* Bock et al. 2010; *Marasphaerium*,* Compactochlorella*,* Masaia* and *Kalenjinia*, Krienitz et al. 2012) were described based on *Dictyosphaerium*‐like algae. Recent investigation of strains with a *Dictyosphaerium*‐like morphology from water bodies of China showed high phylogenetic and morphological diversity of these representatives inside *Parachlorella* clade (Chlorellaceae; Song et al. 2018). Perhaps several new genera of *Dictyosphaerium*‐like algae will be described in the future.

Reports of *Dictyosphaerium* sensu lato in terrestrial habitats are quite limited. The typical aquatic species *Dictyosphaerium pulchellum* (=*Mucidosphaerium pulchellum*) was found in soil occasionally (Ettl and Gärtner 2014). *Dictyosphaerium dichotomum* was described from terrestrial habitats of Antarctica (Ling and Seppelt 1998). The widely distributed *D. chlorelloides* (=*D. minutum*) is typical for aquatic and terrestrial habitats (Komárek and Perman 1978, Tsarenko 2011, Ettl and Gärtner 2014). *Dictyosphaerium terrestre* (Fritsch and John 1942) described from soil of Great Britain is a doubtful taxon and perhaps does not belonged to autospores forming *Dictyosphaerium* sensu lato (Tsarenko and John 2011), because reproduction by zoospores was found (Ettl and Gärtner 2014).

It should be noted that initially *Dictyosphaerium minutum* was described from soil of Denmark (Petersen 1932). It was later transferred to the earlier described species of the aquatic taxon *Dictyosphaerium chlorelloides* because of a very similar morphology (Komárek and Perman 1978). Later investigation of *Dictyosphaerium* based on an integrative approach (Bock et al. 2011a) showed that an aquatic strain characterized by a morphology typical for *Dictyosphaerium chlorelloides* should be assigned to the genus *Chlorella*, and CB 2008/110 was selected as reference strain of *Chlorella chlorelloides*. But strains from terrestrial habitats identified as *D. chlorelloides*/*D. minutum* were not analyzed in this study (Bock et al. 2011a). The new *Xerochlorella* clade includes several strains determined as species of *Dictyosphaerium* found mostly in terrestrial habitats of Europe and Antarctica (see Fig. 1 and Table. 1).

The morphological investigation of the authentic strain *Xerochlorella olmiae* UTEX B 2993 showed characters typical for * Dictyosphaerium chlorelloides*/*D. minutum*: the presence of remnants of mother cell walls in culture and formation of typical 2–4‐celled *Dictyosphaerium*‐like colonies as well as the presence of pyrenoid in cup‐shaped chloroplast. Dissection of the chloroplast on several parts as noted in Fučíková et al. (2014), is due to early stages of autospore formation. The absence of pyrenoid and remnants of the mother cell walls in culture might be explained by the specific state of the respective culture during their investigation which is visible from published micrographs (Fučíková et al. 2014, fig. 1, a–e).

All other strains presented on our phylogenetic trees and attributed to *Xerochlorella* (CCAP 222/3, CCALA 333, UTEX SNO65) are characterized by *Dictyosphaerium*‐like morphology. CCAP 222/3 was initially identified as *Dictyosphaerium minutum* and comprehensively described in Broady (1982). CCALA 333 was identified as another *Dictyosphaerium* species, *D*. *tetrachotomum*, but a micrograph of the species in the collection catalogue showed four‐celled colony typical for *D. minutum* (http://ccala.butbn.cas.cz/en/dictyosphaerium-tetrachotomum-printz). UTEX SNO65 is problematic because it is not available or mislabeled (see Table 1), but according NCBI data this taxon was identified as *Dictyosphaerium* sp. and isolated from Antarctica (https://www.ncbi.nlm.nih.gov/nuccore/GQ502290). Based on all of this information, it is clear that the terrestrial species *D. minutum* is not related to the aquatic taxon *D. chlorelloides* despite close morphology. Furthermore, the genus *Xerochlorella* (type species *X. olmiae*) is in fact synonymous to the widely distributed terrestrial species *D. minutum* discovered more than 80 years ago (Petersen 1932). This taxon is not a species of *Dictyosphaerium* or *Chlorella* (Chlorellaceae), but represents a separate phylogenetic lineage inside the Trebouxiophyceae. Therefore, the validity of the genus *Xerochlorella*, which unites terrestrial *Dictyosphaerium*‐like algae, should be accepted. Assuming *Xerochlorella* as a separate genus, we complemented its diagnosis by new data and proposed respective nomenclatural combination, emendation, and epitypification (see below). *Chlorella umbelloidea* described by Tell (1973) from soil of Argentina is morphologically and ecologically very similar to *D. minutum*, as already mentioned by Komárek and Perman (1978). Therefore, we additionally propose to consider this taxon as a synonym of *Xerochlorella minuta*.

The morphologically different strain Hg‐2‐3 was initially identified as *Dictyosphaerium dichotomum* (Ling and Seppelt 1998, Ettl and Gärtner 2014). Our strain has all characters typical for this representative: formation of large 8‐16‐32(64)‐celled colonies surrounded by mucilage, pseudodichotomous branching of fragments of sporangial walls, typical morphology of single cells. The reproduction of Hg‐2‐3 is usually realized by two autospores, but occasionally by four autospores, the same as indicated by Ling and Seppelt (1998). Large colonies originated due to successive cell division from small two‐celled (Fig. 5, i, k and l) and occasionally four‐celled colonies (Fig. 5, m–o). Phylogenetic analysis showed this strain to belong to the *Xerochlorella* clade, but with a separate lineage. Therefore, we assume that this strain represents a separate species of *Xerochlorella*. Thus the respective taxonomic combination and epitypification of this taxon is proposed (see below).

### Morphological characters of *Xerochlorella*

Morphological parallelism is a phenomenon widely distributed among algae. Perhaps morphological similarity of the freshwater *Chlorella chlorelloides* (Chlorellaceae) and the terrestrial *Xerochlorella minuta* (separate lineage in Trebouxiophyceae, Trebouxiophyceae incertae sedis; Guiry and Guiry 2019) is an example of such morphological parallelism. But also some morphological characters specific for each taxon were found. A pyrenoid surrounded by 2(4) starch grains is typical for representatives of Chlorellaceae including *Chlorella chlorelloides* (Bock et al. 2011a, Ettl and Gärtner 2014). TEM micrographs of the investigated *Xerochlorella* strains showed the presence of a pyrenoid surrounded by several (5–6) starch grains (see Fig. 7). Similar structure of pyrenoid with numerous starch grains was also observed by Broady (1982, figs. 66–67) in *Dictyosphaerium minutum* (now *X. minuta*) isolated from Antarctica. A similar pyrenoid structure was found in *D. chlorelloides*, isolated from granite outcrops of the South of Ukraine (Mikhailyuk and Demchenko 2005).

Another typical morphological character of *Xerochlorella* is its specific structure of mother (sporangial) cell wall fragments, which form a *Dictyosphaerium*‐like morphology. Cells of *Dictyosphaerium*‐like algae from the Chlorellaceae are connected to thin mucous strands, which originated from mother (sporangial) cell wall fragments (Bock et al. 2011a). Analogical structures of *Xerochlorella* completely maintain the initial semilunar or cruciform shape of ruptured sporangial walls and do not turn to mucous strands. This was already mentioned in the first description of *D. minutum* (Petersen 1932) and during observation of this species by Broady (1982). Ling and Seppelt (1998) also noted as one specific character of *D. dichotomum*: “mother cell wall fragments do not change into mucous strands as is often the case in *Dictyosphaerium*” (p. 59).

The presence of a common mucilage envelope surrounding colonies in *Xerochlorella* is a disputable question. Mucilage envelope and colonies were not observed by Fučíková et al. (2014) in *X. olmiae* strains. Petersen (1932) did also not observe common mucilage envelope in *Dictyosphaerium minutum*, although mentioned slight gelatinization of cell walls. Komárek and Perman (1978), however, noted different development of a mucilage envelope in *D. chlorelloides*: from implicit or slight mucilage to prominent strong envelope sometimes with clear individual envelopes surrounding cells. A common mucilage envelope of 3 μm thickness after negative staining with ink was indicated for *D. dichotomum* (Ling and Seppelt 1998, figs. 13, 20). Our observations showed also different phenotypic expression of a common mucilage envelope in strains of *X. minuta* as well as the presence of individual envelopes surrounding cells (see Fig. 6). Negative staining of strain Hg‐2‐3 confirmed the presence of a clear common mucilage envelope with a thickness of 2.1‐3.0 μm. Therefore, members of *Xerochlorella* have a common mucilage envelope with different thickness depending on environmental conditions. This mucilage envelope is usually thin, delicate, homogeneous, and start to be visible after negative staining by ink.

### Ecology and distribution of *Xerochlorella*

Terrestrial *Dictyosphaerium* (*Xerochlorella minuta*) are widely distributed species. Ettl and Gärtner (2014) noted its wide distribution in Europe (Denmark, Great Britain, Iceland, former USSR) and Asia and some findings in Antarctica. This taxon was mentioned in main check‐lists and hand books as widely distributed terrestrial alga (Komárek and Fott 1983, Andreyeva 1998, Tsarenko and John 2011). We found it repeatedly as terrestrial alga from granite outcrops of the South of Ukraine (Mikhailyuk and Demchenko 2005, Mikhailyuk et al. 2011) as well as in biological soil crusts from sand dunes of Germany and Ukraine (Mikhailyuk et al. 2019), forests of Germany (Glaser et al. 2018) and polar regions such as the Antarctic Peninsula and Arctic Svalbard (Borchhardt et al. 2017a,b).

Molecular‐phylogenetic data were obtained from *Xerochlorella minuta* strains isolated from terrestrial and amphibian (swamp) habitats of Western and Eastern Europe (Germany, Ukraine, Slovakia), North America (USA), and Antarctica (see Table 1). These data prove that all investigated strains are genetically very close despite distant geographical regions and different ecological conditions (from swamps and moist soils to maritime sand dunes, habitats in hot and cold deserts). All these strains can be considered as representatives of different populations of the same species. Therefore, it is reasonable to assume that *X. minuta*, due to its small cell size and possible high tolerance to temperature and lack of water, can be easily distributed by wind (or other vectors) over long distances which is typical for terrestrial algae (Sharma et al. 2007, Ryšanek et al. 2015). Despite the fact that this species was originally described from Western Europe we propose the American strain (authentic strain of *Xerochlorella olmiae;* see below) as reference strain of *X. minuta*.

The other species of *Xerochlorella*,* X. dichotoma*, was originally described from soils of Antarctica. Cultures were used for species description, but all strains were lost during transportation of the material from Antarctica to Australia (Ling and Seppelt 1998). We do not know other findings of this taxon next to the type locality. Our strain, identified as *X. dichotoma*, was isolated from maritime sand dunes, Germany (Table 1). Despite the fact the type locality of *X. dichotoma* is situated far away from the Baltic Sea, we assume that strains from both regions are representatives of the same species, and hence expect the same cosmopolitan biogeographical distribution as for *X. minuta*. But it is obvious that *X. dichotoma* is a rare species. Based on all this information we propose the here investigated strain as reference of *X. dichotoma*, taking into consideration its complete morphological similarity with the original species description (formation of large colonies surrounded by mucilage, pseudodichotomous branching of fragments of sporangial walls, typical morphology of single cells), close ecological characteristic (sand vs. sandy soils) and cosmopolitan distribution of the genus *Xerochlorella*.

### Proposed taxonomic changes

***Xerochlorella*** Fučíková, P.O. Lewis & L.A. Lewis (2014). *Phycological Research* 62:304, fig. 1, a–e. emend. Mikhailyuk & P.M. Tsarenko

*Emended diagnosis*: Cells solitary or in colonies surrounded by delicate mucilage envelopes. Cells ovoid to wide ellipsoid and almost spherical, thin‐walled, uninucleate, connected to fragments of mother (sporangial) walls with their narrow ends (*Dictyosphaerium*‐like morphology). Chloroplasts single, parietal, cup‐shaped, with pyrenoid surrounded by several starch grains. Asexual reproduction via autospores. Autospores released by rupture of sporangial wall and further preservation of fragments with connected cells. Sexual reproduction not observed.

Type species: *Xerochlorella minuta* (J.B. Petersen) Mikhailyuk & P.M. Tsarenko comb. nova

***Xerochlorella minuta*** (J.B. Petersen) Mikhailyuk & P.M. Tsarenko comb. nova (Figs. 5, a–g; 6, a–e; 7, a–d)

*Basionym*:* Dictyosphaerium minutum* J.B. Petersen (1932) *Bot. Tiddskr*. 42:37, fig. 19.

*Synonyms*:* Chlorella umbelloidea* Tell, *Xerochlorella olmiae* Fučíková, P.O. Lewis & L.A. Lewis, as “*olmae*” (according to Art. 60.8 of the ICN [Turland et al. 2018], the original spelling “olmae” is corrected to “olmiae”).

*Emended diagnosis*: Cells solitary or in small 2‐4‐celled (sometimes to 16‐celled) colonies surrounded by delicate mucilage envelope (2.1–5.0 μm thickness). Cells small, ovoid to wide ellipsoid and almost spherical, (4.3)5.0–6.7(7.5) × (2.5)4.3–6.0(7.0) μm, connected to semilunar or cruciform fragments of mother (sporangial) walls with their narrow ends. Chloroplasts single, parietal, cup‐shaped, occupied half or 2/3 of cells inner surface, with pyrenoid surrounded by several (5–6) starch grains. Asexual reproduction via 2–4 autospores released by rupture of sporangial wall. Zoospore formation and sexual reproduction not observed.

*Type locality*: soil, beech copse, Hammer Bakker, Denmark.

*Lectotype* (designated here): fig. 19 in Petersen 1932.

*Epitype* (designated here)*:* culture material of the strain UTEX B 2993 preserved as specimen CONN00178617 (Fučíková et al. 2014) that is supported by the lectotype.

*Reference strain*: UTEX B 2993

Comments: The reference strain completely corresponds to the diagnosis of *Dictyosphaerium minutum* (Petersen 1932). The species diagnosis was supplemented by the details of pyrenoid structure and refined dimensional ranges for cells. The reference strain was isolated from desert biological soil crust (Mojave National Preserve, San Bernardino Co., California, USA) and represented the authentic strain of *Xerochlorella olmiae* (Fučíková et al. 2014). This habitat is ecologically similar, but geographically distant from the type locality (soil, Denmark). But since *Xerochlorella* species are widely distributed over the world, as was shown in the present study, and strain UTEX B 2993 is phylogenetically close to European strains, it is proposed as a reference strain.

***Xerochlorella dichotoma*** (H.P. Ling & R.D. Seppelt) Mikhailyuk & P.M. Tsarenko comb. nova (Figs. 5, h–o; 6, f–k; 7, e–j)

*Basionym*:* Dictyosphaerium dichotomum* H.P. Ling & R.D. Seppelt (1998). Arch. Hydrobiol. Suppl. 124 (Algol Stud. 89):57, figs. 13–17.

*Emended diagnosis*: Cells solitary or in large (2‐4)‐8‐16‐32‐(64)‐celled colonies surrounded by delicate mucilage envelope (2.1–3 μm thickness). Cells connected to semilunar or cruciform fragments of mother (sporangial) walls with their narrow ends. Subsequent cell divisions form pseudodichotomous branching. Cells small, ovoid to wide ellipsoid and almost spherical, (4.5)6.1–8.1(9.6) × (3.9)5.7–7.8(8.6) μm. Chloroplasts single, parietal, cup‐shaped, occupied half or 2/3 of cells inner surface, with pyrenoid surrounded by several (5–6) starch grains. Asexual reproduction mostly via 2, occasionally via 4 autospores, released by rupture of sporangial wall. Zoospore formation and sexual reproduction not observed.

*Type locality*: sandy soil, Stevenson Cove, Casey, Antarctica.

*Lectotype* (designated here): figs. 13‐17 in Ling & Seppelt (1998).

*Epitype* (designated here)*:* preserved specimen 160223‐7, fixed for TEM embedded material of the strain Hg‐2‐3 (SAG 2582) (Department of Botany, Innsbruck University). Additionally, KW‐A‐32502, preserved culture material of reference strain Hg‐2‐3 (SAG 2582), Algotheca, Herbarium of the M.G. Kholodny Institute of Botany of the National Academy of Sciences of Ukraine (KW).

*Reference strain*: Hg‐2‐3 was deposited in the Sammlung von Algenkulturen, University of Göttingen, Germany, under number SAG 2582.

*Comments*: The reference strain completely corresponds to the diagnosis of *Dictyosphaerium dichotomum* (Ling and Seppelt 1998). The species diagnosis was supplemented by the details of pyrenoid structure and refined dimensional ranges for cells and colonies. The strain was isolated from biological soil crusts on maritime sand dunes, coast of the Baltic Sea, Heiligendamm, Mecklenburg‐Vorpommern, Germany. This habitat is ecologically similar, but geographically distant from the type locality (sandy soil, Antarctica). But since *Xerochlorella* species are widely distributed over the world, as was shown in the present study, the strain from Europe is proposed as reference.

### Proposed nomenclatural changes for *Dictyosphaerium chlorelloides*

During this investigation some inaccuracy on the taxon *Dictyosphaerium chlorelloides* (=*Chlorella chlorelloides*) was detected. Taxonomic combination *D. chlorelloides* provided *Brachionococcus chlorelloides* as basionym, published by Naumann in 1919 with figures (holotype), belonging to the work from 1921 (Komárek and Perman 1978, p. 252). Naumann's work was cited in this reference list as published in 1919. The same information was repeated in the paper that provided taxonomic combination of *Ch. chlorelloides*, without any notion of Naumann's work in the reference list (Bock et al. 2011a). *Brachionococcus chlorelloides* is mentioned among Internet sources as taxon published in Naumann (1919) together with figures (http://ucjeps.berkeley.edu/ina/). However, investigation of the respective literature showed that Naumann's paper was submitted in 1919, but in fact volume 16(2) of Arkiv för Botanik was published in February 1921. The errors in citation of the basionym do not preclude valid publication of new combinations (Art. 41.6 of the ICN; Turland et al. 2018). Therefore, this inaccuracy was corrected and all previous taxonomic combinations of *Brachionococcus chlorelloides* were kept (see below).

*Chlorella chlorelloides* (Naumann) C. Bock, Krienitz & Pröschold

*Basionym*:* Brachionococcus chlorelloides* Naumann (1921). *Ark. Bot*. 16(2):15, figs. 8–9.

*Synonym*:* Dictyosphaerium chlorelloides* (Naumann) Komárek and Perman (1978)

This study was supported by a Georg‐Forster research fellowship from the Alexander von Humboldt Foundation (T.M.). The work has been funded by the DFG Priority Program 1374 “Infrastructure‐Biodiversity‐Exploratories” (subproject Crustfunction‐KA899/28‐1, U.K.), by the Priority Program 1991 Taxonomics (GL 909/1‐1, K.G.) and by the FWF grant I 1951‐B16, A.H. The study was summarized during a stay of T.M. at the University of Innsbruck, Austria funded by a program to revise the culture collection of alpine algae (ASIB) at the Institute of Botany. We thank the managers of the three Biodiversity Exploratories, Martin Gorke, and all former managers for their work in maintaining the plot and project infrastructure, Christiane Fischer for giving support through the central office, Michael Owonibi for managing the central database, and Markus Fischer, Eduard Linsenmair, Dominik Hessenmöller, Daniel Prati, Ingo Schöning, François Buscot, Ernst‐Detlef Schulze, Wolfgang W. Weisser, and the late Elisabeth Kalko for their role in setting up the Biodiversity Exploratories project. Fieldwork permits were issued by the responsible state environmental offices of Brandenburg (according to §72 BbgNatSchG). Our sincere thanks are extended to Sabrina Obwegeser and Beatrix Jungwirth, University of Innsbruck, Austria, for providing help in the TEM investigations. We are grateful to Sergei L. Mosyakin (M.G. Kholodny Institute of Botany, NAS of Ukraine) for his valuable advices on nomenclatural issues and Dr. Maike Lorenz, University of Göttingen, for help during strain deposition to SAG.
